# Radon Exhalation Rate: A Metrological Approach for Radiation Protection

**DOI:** 10.3390/s24113633

**Published:** 2024-06-04

**Authors:** Fabrizio Ambrosino, Giuseppe La Verde, Gaetano Gagliardo, Rocco Mottareale, Giuseppe Della Peruta, Chiara Imparato, Andrea D’Elia, Mariagabriella Pugliese

**Affiliations:** 1 Department of Physics “E. Pancini”, University of Naples Federico II, 80126 Naples, Italy; fabrizio.ambrosino@unina.it (F.A.); mpuglies@na.infn.it (M.P.); 2 National Institute of Nuclear Physics (INFN), Naples Section, 80126 Naples, Italy

**Keywords:** radon exhalation and emanation, building materials, indoor air quality, alpha spectrometry, metrology, living environment

## Abstract

Radon, a radioactive inert gas that comes from the decay of naturally occurring radioactive species, poses a substantial health risk due to its involvement in lung cancer carcinogenesis. This work proposes a metrological approach for determining radon exhalation rates from diverse building materials. This methodology employs an electrostatic collection chamber for alpha spectrometry of radon isotopic decay products. Experimental evaluations were conducted particularly focusing on volcanic gray tuff from Sant’Agata de’ Goti (Campania region, Italy), a material commonly utilized in construction, to assess radon exhalation rates. The study aligns with Legislative Decree 101/2020, a transposition of European Directive 59/2013/Euratom, highlighting the need to identify materials with a high risk of radon exhalation. Moreover, this work supports the goals of the Italian National Radon Action Plan related to the aforementioned decree, aiming to develop methodologies for estimating radon exhalation rates from building materials and improving radioprotection practices.

## 1. Introduction

Every building material (BM) contains various concentrations of naturally occurring radioactive isotopes. Materials derived from rock and soil primarily contain natural radionuclides from the uranium (^238^U) and thorium (^232^Th) decay series as well as the radioactive potassium isotope (^40^K) [1]. Radiation exposure from building materials (BMs) can be classified into external and internal exposure. The aim behind regulating the radioactivity of BMs is to restrict radiation exposure arising from materials exhibiting increased or heightened levels of natural radionuclides [2]. The average yearly duration of occupants’ exposure to natural ionizing radiation within buildings amounts to 7000 h, whereas public exposure to radiation from BMs used in alternative structures like tunnels, bridges, etc., is notably briefer [3]. Among the various naturally occurring radionuclides included in BMs, radon plays a crucial role [4,5]. Radon is a radioactive inert gas originating from the decay of naturally occurring radioactive species, such as ^238^U and ^232^Th, found in minerals and crustal rocks, resulting in the production of radioisotopes, including ^222^Rn (half-life 3.82 days) and ^220^Rn (half-life 55.6 s). Radon isotopes tend to accumulate in poorly ventilated areas because of their density, which makes them heavier than air. Migrating through the soil via pores, faults, and fractures, radon isotopes gradually rise to the surface atmosphere and then seep into enclosed indoor spaces [6,7]. Radon exposure poses a significant health risk due to its direct contribution to carcinogenesis [8,9,10] through the emission of high LET alpha particles [11] and/or the subsequent decay of its daughter nuclides following inhalation [12]. The respiratory tract serves as the primary route of radon entry into the organism, where radon exposure has been firmly linked to lung cancer development [13,14]. For this reason, radon has been classified as a Group I carcinogen radionuclide by the International Agency for Research on Cancer [15]. Moreover, the World Health Organization (WHO) identified radon presence in indoor air as a major risk agent for the probability of developing lung cancer, contributing to an estimated proportion of global lung cancer cases ranging from 3% to 14% [16]. In Italy, considering the existence of such a sanitary risk associated with radon exposure and inhalation, Legislative Decree 101/2020 (L.D. 101/2020) [17] established a reference level for the annual average activity concentration of indoor radon at 300 Bq/m^3^. L.D. 101/2020, implementing European Directive 59/2013/Euratom [18], lays down basic safety standards for protection against dangers arising from exposure to ionizing radiation. In particular, Annex II of L.D. 101/2020 provides further guidance on the identification of building materials (BMs) for investigation. Specifically, it outlines a categorized list of BMs, which includes (i) natural materials, such as alum-shale and BMs or additives derived from natural igneous sources, like granitoids, porphyry, tuff, pozzolan, lava, and derivatives of zirconiferous sands, and (ii) materials incorporating residues from industries processing naturally occurring radioactive materials (NORM), including fly ash, phosphogypsum, phosphorus slag, tin slag, copper slag, red mud, and residues from steel production. In terms of radon exhalation radiological risk management, the Decree also requires the introduction of the National Radon Action Plan (NRAP) published on 21 February 2024 [19], which points out the importance of identifying building materials (BMs) and the related radon exhalation rate. In particular, Action 2.3 of the NRAP aims to identify and classify BMs with higher radon exhalation rates providing tools for their appropriate use, both structural and architectural, from production to delivery on site and final incorporation into the work. The determination of this parameter is extremely important for the evaluation of radon flux toward indoor air and hence the indoor radon hazard. In this regard, the NRAP highlights the need for the development of a methodology to estimate the rate of radon exhalation from BMs. As discussed, BMs employed in building constructions stand as a significant source of radon, notably in historic villages, such as Sant’Agata de’ Goti, Italy, entirely constructed from tuff, a material acknowledged for its elevated natural radionuclide content. Within this framework, the necessity to preserve artistic heritage and protect public health by examining the radiological composition of such materials arises as the central focus of this study. Furthermore, it is of crucial importance to compare different commonly used BMs. In this framework, the current work proposes a metrological approach for the determination of radon exhalation rates from different matrices of materials employed in building constructions [20]. The methodology involves the development and optimization of an electrostatic collection chamber for alpha spectrometry of radon isotopic decay products. The experimental setup was tested for the measurement of exhalation rates from volcanic grey tuff with high radon activity concentrations commonly and extensively employed in buildings of the Italian village Sant’Agata de’ Goti in Campania. The values obtained for the Sant’Agata de’ Goti Tuff were compared with those documented in the literature for analogous types of grey tuffs from the Campania region. This approach aimed to provide an effective contribution to the radon exhalation risk management in BMs that can be utilized for radioprotection practices by competent national authorities in agreement with the NRAP.

## 2. Materials and Methods

Radon, a noble gas, emanates into the environment and can be inhaled, posing a significant health hazard. In this context, determining the concentrations of radon emitted from BM is of utmost significance. Indeed, BMs with similar activity concentrations of radionuclides, porosities, and diffusion coefficients can have significantly different emanation coefficients and generate radon fluxes of different magnitudes [21]. To determine the radon exhalation rate of the tested tuff sample collected in the Italian village of Sant’Agata de’ Goti (Campania), an experimental apparatus was developed and optimized. Specifically, since gamma spectrometry alone cannot assess the emanation capacities, which depend on natural radionuclide concentrations and petrographic factors, this method combines it with radon emanation measurements obtained through an alpha spectrometer electrode within an electrostatic collection chamber. In detail, gamma spectrometry was employed to obtain ^226^Ra and ^232^Th activity concentrations, while alpha spectrometry allowed the assessment of ^222^Rn and ^220^Rn isotope activity concentrations per unit volume (see Section 2.2). Both types of data enabled the quantification of radon isotope emanation and were exploited for the calculation of radon exhalation rates. The system was assembled and tested at the LaRa Radioactivity Laboratory of the Department of Physics “Ettore Pancini” at the University Federico II of Naples. Performing gamma spectrometry on BMs is crucial for two reasons. Firstly, the concentrations of ^226^Ra and ^232^Th are essential for determining their respective emanation coefficients (Formulas (1) and (3)). Moreover, external exposure from BMs is extensively regulated by legislative directives (European Directive 59/2013/Euratom and Italian Legislative Decree 101/2020). The reference level for indoor gamma radiation exposure emanating from BM, alongside outdoor exposure, is established at 1 mSv/year. However, the initial step involves utilizing a screening tool known as Index I (outlined in Annex II of L.D. 101/2020), which, based on the activity concentrations of ^226^Ra, ^232^Th, and ^40^K, allows for the preliminary identification of building materials with radioprotection significance. In our study, we primarily focused on refining a metrological approach to determine radon exhalation rates and emanation coefficients from different matrices of building materials. In this context, determining concentrations through gamma spectrometry is of crucial importance. Nonetheless, for risk management procedures regarding external exposures from construction materials, see [1,21,22].

### 2.1. Electrostatic Collection Chamber

**Figure 1:**
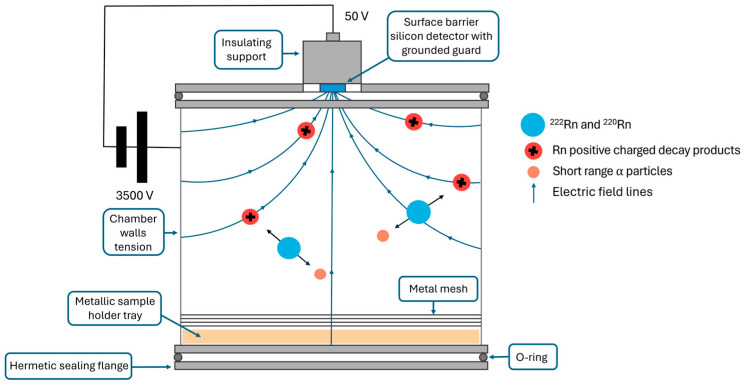
Schematic representation of the electrostatic collection chamber.

The electrostatic collection chamber, fine-tuned and calibrated, consists of a cylindrical steel chamber with a diameter and a length of 10 cm, hermetically lockable by two flanges made of the same material. The volume of the chamber is about 0.8 L, and at the bottom, a metal tray holds the sample for analysis, covered by a tightly meshed metal grid adhering to the cylinder’s wall to ensure a homogeneous electric field. At the center of the upper flange, a surface barrier silicon detector from Ametek (model BU 019300100) for alpha particle detection is placed on an insulating support. The detector is electrically insulated from the entire chamber, which is under a voltage of 3500 V. From measurements conducted during the calibration phase of the chamber, at various voltage values (ranging from 0 to 3500 V), it was observed that the counts related to ^218^Po increased with increasing voltage. Therefore, the electrostatic collection efficiency varies as a function of the voltage applied to the chamber walls and is maximal for values of the electric field intensity, ensuring the complete collection of all charged radionuclides generated within the chamber before their decay or neutralization. The limit in our case was imposed by uncontrolled discharge phenomena occurring at a voltage of 4000 V. The apparatus is in Figure 1.

According to the manufacturer’s specified operating conditions, the detector has a maximum nominal resolution of 19 keV (FWHM) for alpha particles with an energy of 5.846 MeV emitted from the decay of ^241^Am and 14 keV (FWHM) for beta particles. The active surface area is 300 mm^2^, and the minimum depth of the depleted sensitive zone is 100 μm. The detector, electrically insulated from the rest of the chamber through a suitable support, is subjected to a polarization potential of 50 V during the measurements. The experimental setup, in addition to the electrostatic collection chamber, includes:

- a high-voltage power supply for electrostatic collection;
- a power supply for the polarization voltage of the detector;
- a charge-sensitive preamplifier;
- a linear amplifier;
- a multichannel analyzer.

The decay products of ^222^Rn and ^220^Rn are positively charged ions; the recoil resulting from the emission of the alpha particle by radon and the sudden modification of the nuclear electrostatic field cause the loss of some electrons from the formed decay products. Inside the collection chamber, the radon decay products are directed toward the surface of the detector by the chamber electrostatic field, where they further decay by emitting an alpha particle. The detector’s excellent energy resolution and the lack of energy degradation of alpha particles emitted by radionuclides allow for the precise separation of peaks related to different decay products of ^222^Rn and ^220^Rn. The approach of separating ^220^Rn and ^222^Rn allows for a more accurate assessment of the health risks associated with these two radionuclides; specifically, although ^220^Rn has a neglectable half-life (of only a few seconds) in terms of directly associated radiological risk, it acts as an indicator of the concentration of Th-232, its progenitor. Therefore, as also required by the NRAP [19], in terms of correctly evaluating the air quality in indoor environments characterized by radon issues, the assessment of the two radionuclides must be conducted separately. In detail, the multichannel analyzer provides the complete alpha spectrum of radon decay products, including the two lines of polonium radioisotopes, ^218^Po at 6.01 keV and ^214^Po at 7.68 keV for ^222^Rn, and the lines of ^216^Po at 6.77 keV, ^212^Bi at 6.09 keV, and ^212^Po at 8.77 keV for ^220^Rn. The areas of these peaks can be used, along with an appropriate calibration factor (see Section 2.3), for the calculation of the concentrations of ^222^Rn and ^220^Rn, after reaching the equilibrium with their progenitors. As the chamber is hermetically sealed, the radon emitted by the sample under examination is confined within the free volume of the container. Its concentration increases from zero at the moment of chamber closure to the maximum value upon reaching equilibrium between the emanating radon and the decaying radon progenies.

### 2.2. Emanation and Exhalation Rate

The emanating radon is the fraction of radon atoms preserving enough kinetic energy to leave the grain of the material where it has been generated and reach the empty space in the porous materials. The exhaled radon is the fraction of radon atoms reaching the pore volume of the material mass that can escape into the air outside the material and diffuse into the spaces where people live [23]. 

The emanation coefficient is defined as the ratio between the quantity of radon emitted within the porosity of the material and the total quantity of radon contained in the sample. Considering the experimental setup, the emanation coefficient *η* of the ^222^Rn isotope from the sample was calculated using the following formula [24]:

(1) $$\eta_{222_{Rn}}=\frac{C_{222_{Rn}\;ema}}{C_{226_{Ra}}}\cdot \frac{V}{m}$$

$C_{222_{Rn}\;ema}$ represents the activity concentration (Bq/m^3^) per unit volume measured using alpha spectroscopy, $C_{226_{Ra}}$ is the activity concentration of radium in the sample (Bq/kg) from gamma spectrometry (see Section 2.5), *V* is the volume of the chamber (m^3^), and *m* is the mass of the sample (kg). To measure $C_{222_{Rn}\;ema}$, the procedure was carried out using alpha spectroscopy through the following relationship:

(2) $$C_{222_{Rn}\;ema}=\frac{{cps}_{218_{Po}}}{\varepsilon_{218_{Po}}}$$

where ${cps}_{218_{Po}}$ represents the counts per second of alpha particles emitted by ^218^Po and detected on the silicon, while $\varepsilon_{218_{Po}}$ is the collection efficiency of ^218^Po obtained through appropriate calibration (see Section 2.3).

Similarly, the same discussions can be applied to ^220^Rn, resulting in the following relationships:

(3) $$\eta_{220_{Rn}}=\frac{C_{220_{Rn}\;ema}}{C_{232_{Th}}}\cdot \frac{V}{m}$$

(4) $$C_{220_{Rn}\;ema}=\frac{{cps}_{216_{Po}}}{\varepsilon_{216_{Po}}}$$

The exhalation rate *E* is defined as the quantity of radon released from the sample per unit of time. Assuming that E is constant over time (a valid assumption when the chamber volume is much larger than the sample volume), and under the assumption that the sample thickness is negligible compared with the mean free path of radon diffusion within it avoiding the back diffusion process [24], the ^222^Rn and ^220^Rn the exhalation rates are (where *λ* is the decay constant):

(5) $$E_{222_{Rn}}=C_{226_{Ra}}\cdot \eta_{222_{Rn}}\cdot \lambda_{222_{Rn}\;}$$

(6) $$E_{220_{Rn}}=C_{232_{Th}}\cdot \eta_{220_{Rn}}\cdot \lambda_{220_{Rn}\;}$$

### 2.3. Calibration

**Figure 2:**
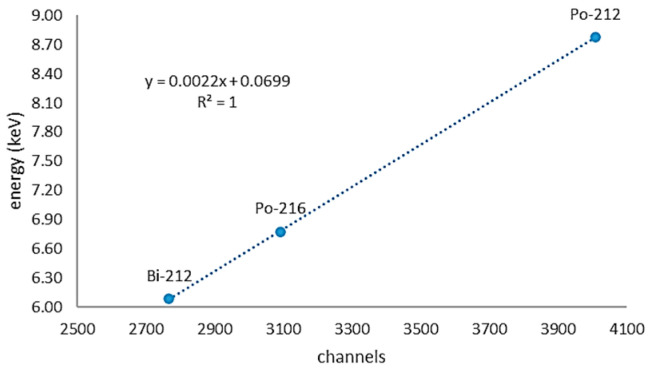
Channels associated with the energy peak values of the ^220^Rn ionic progeny. The equation of the curve is also reported in the figure.

In order to obtain the energy spectrum necessary for counting the alpha decays of the progeny of ^222^Rn and ^220^Rn, a source containing thorium salts, Th(NO_3_)_4_, was introduced. Thanks to the energy resolution of the silicon detector, peaks corresponding to the decay of the ^220^Rn ionic progeny were identified and associated with a channel of the multichannel analyzer (MCA). In Figure 2, the correspondence between the energy of alpha particles emitted from the decay of radionuclides and the associated channel is reported.

**Figure 3:**
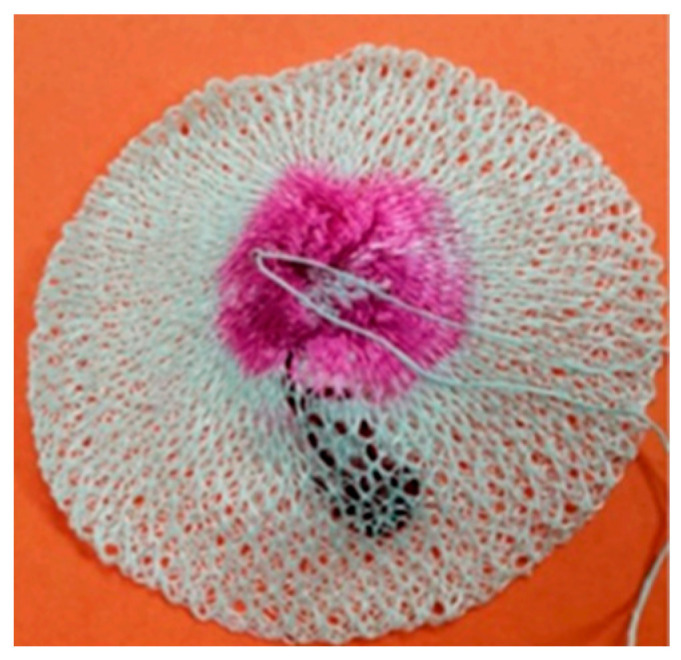
Thorium-containing incandescent lamp mantle.

To calibrate the alpha spectroscopy apparatus regarding the decay products of ^220^Rn, the same source of thorium nitrate Th(NO_3_)_4_ at a known activity was used. As shown in Figure 3, a mantle containing ^232^Th typically used for incandescent lamps was employed. The mantle with Th salts was placed in the sample holder tray of the electrostatic chamber and used subsequently to measure the activity concentration of the analyzed tuff sample (see Figure 1). From the mantle, ^220^Rn naturally exhalates, and its ionized decay products reach the detector. The ^216^Po counting efficiency is equal to:

(7) $$\varepsilon_{216_{Po}}=0.093\;\pm \;0.005\;cps\cdot L/Bq\;$$

**Figure 4:**
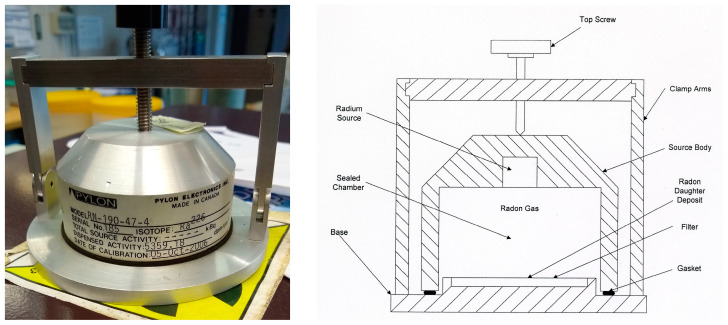
Pylon RN-190 radon source (**left**) and schematization of the internal structure (**right**).

The calibration of the alpha spectroscopy apparatus regarding the decay products of ^222^Rn was performed using a standard source of ^226^Ra from Pylon, model RN-190, Ottawa, ON, Canada [25] (Figure 4). As shown in Figure 4, a pure ^226^Ra source is hermetically sealed inside a structure equipped with a screw pin to ensure a tight seal.

The ^226^Ra source has a half-life of 1600 years and is designed to emit 100% of the ^222^Rn uniformly and independently of environmental conditions. The calibration effectiveness is not affected by humidity levels, dust, temperature, or the growth of ^222^Rn decay products within the chamber walls. The concentration of ^222^Rn, produced in the gaseous state through the decay of ^226^Ra, begins to increase inside the chamber once it is hermetically sealed, reaching a saturation value. By inserting a specific paper filter inside the structure for a certain period, it absorbs ^222^Rn atoms and its decay products. After a minimum waiting time of 24 h, the activity concentration level deposited on the filter becomes proportional to the ^222^Rn concentration inside the chamber. The ^222^Rn activity level on the filter tends to increase over time while the chamber is sealed, then decreases upon opening following the laws of radioactive decay. By noting the time t_0_ when the filter is inserted into the sealed Pylon chamber, the time t_1_ when the filter is removed from the sealed chamber, the time t_2_ when the measurement of the filter activity concentration in the ion collection chamber begins, and the time t_3_ when the counting ends, the efficiency of the apparatus chamber can be calculated using the following formula [26]:

(8) $$\varepsilon =\frac{C(t_{2},t_{3})}{Q_{0}\cdot R(t_{1})\cdot M(t_{2},t_{3})\cdot A}$$

where:

$C(t_{2},t_{3})$ is the number of counts between time *t*_2_ and *t*_3_ (125 s);

*Q*_0_ is the number of decays per minute (5359.18 dpm/cm^2^);

*R*(*t*_1_) is an equilibrium factor given by the formula:

(9) $$R\left(t_{1}\right)=1-e^{-\lambda (t_{1}-t_{0})}$$

$M(t_{2},t_{3})$ is a coefficient provided by Pylon depending on the start time and duration of the calibration measurement [25];

A is the surface area of the filter expressed in cm^2^ (4.9 ± 0.1 cm^2^).

Therefore, the following procedure was carried out: the Pylon chamber was opened by unscrewing the upper pin until it was possible to laterally bend the arms, ensuring the seal. The paper filter was inserted, the chamber was closed, and the start time *t*_0_ of the filter exposure to the radio source was noted. The filter was left in the chamber for at least 24 h. The filter was extracted from the chamber, and the time t_1_ of extraction was noted. The equilibrium factor *R*(*t*_1_) was calculated using formula (9). The filter was inserted into the electrostatic collection chamber, and the start time *t*_2_ of the activity concentration measurement was noted. The end time t_3_ of the measurement, which should last at least 5 min, was noted. The measured concentration value, equilibrium factor *R*(*t*_1_), and the values of times t_2_ and t_3_ were used to calculate the efficiency using formula (8).

The value thus obtained for the electrostatic collection efficiency relative to ^218^Po is:

(10) $$\varepsilon_{218_{Po}}=0.36\;\pm \;0.01\;cps\cdot L/Bq\;$$

### 2.4. Sampling

In this study, the results include measurements of the emanation coefficient and exhalation rate for volcanic gray tuff samples sourced from Sant’Agata de’ Goti village in Campania, Italy. The methodology outlined in UNI EN ISO 18589–2:2015 [27] was adopted for sample preparation to ensure a consistent and stable matrix. This approach ensures sample uniformity and homogeneity. Bricks were ground into powder using a grinder (PM 100 Retsch, Haan, Germany), sieved, and then dried in an oven (DIGITRONIC Selecta 2005141, Barcelona, Spain) at 105 °C for two hours. The powder was subsequently homogenized. The final product was weighed and enclosed in a Marinelli Becker container for a period of 4 weeks, allowing for the attainment of secular equilibrium between ^226^Ra and its gamma-emitting daughters.

The geological formation supporting the village of Sant’Agata de’ Goti comprises Campanian gray tuff, a result of the eruption of the Campanian ignimbrite around 39,395 years ago [28]. 

The Campanian gray tuff possesses a trachytic composition characterized by a matrix deposit comprising biotite, along with porphyritic black pumices containing sanidine. Additionally, it contains occasional gray scoriae with large sanidine phenocrysts as well as inclusions of lava fragments and sedimentary substrate. The dominant crystals found within the matrix consist mainly of scattered sanidine crystals, alongside plagioclase, biotite, and clinopyroxene. The bottom layer of the Campanian gray tuff, forming the fallout layer, consists of alkaline trachyphonolitic pumices [29]. As one ascends from the lowest level, the occurrence and dimensions of dark pumices progressively increase, while the presence of lava and sedimentary fragments diminishes. The highest section of the ignimbrite, being the most exposed, might display a yellowish hue. This yellow appearance, differing from the Neapolitan yellow tuff formed by an eruption 15,000 years ago, gains its color and enhanced texture from a secondary lithification process, often associated with zeolitization [30]. The majority of the village’s buildings are founded directly on the tuff layer and are built using tuff bricks, a natural building material rich in radionuclides, sourced from the underlying Campanian ignimbrite layer. A technique widely practiced and documented in [31] results in the creation of a distinctive landscape: there are 160 underground cavities at the level of this layer [32]. The geological and lithological makeup renders the village of Sant’Agata de’ Goti susceptible to various factors that elevate indoor radon concentrations. Alongside the underlying igneous formations, the prevalent use of tuff as a building material for most structures, and the underground voids facilitating gas accumulation, additional factors like the ventilated climate promoting wind circulation and the presence of tuffaceous peaks aiding gas release, further support the notion that the local population might face an elevated radon risk. A significant amount of information regarding the subsurface of Sant’Agata de’ Goti stems from a study conducted in 1994, which included the drilling of 40 deep boreholes reaching depths of up to 40 m [32]. The tuffaceous summit overlays a flysch-like deposit layer composed of Miocene terrigenous sequences emerging from the Martorano Valley [33]. This layer is primarily composed of sandy–clayey sediments. The layer that follows comprises Campanian ignimbrite, originating from an eruption of the Campi Flegrei 39,000 years ago [28,34]. In sequence, this layer contains two primary lithofacies: a deeper and considerably more prevalent layer of gray facies Campanian ignimbrite, referred to as Welded Gray Ignimbrite (WGI), and a shallower layer of yellow facies Campanian ignimbrite, known as Lithified Yellow Tuff (LYT) [30]. The WGI, having undergone autogenic feldspathization, presents a pozzolanic and pumiceous matrix in its deeper layers, transitioning to a cineritic nature in the upper layers. Conversely, the LYT comprises a cineritic matrix containing rounded lapilli and pumiceous clasts. The uppermost layer comprises several meters of pyroclastic soil and backfill terrain, known as Made Ground-Pyroclastic Soil (MG-PS) [32].

### 2.5. Gamma Spectrometry

High-resolution gamma spectrometry was conducted employing a coaxial High-Purity Germanium (HPGe ORTEC^®^) detector (model GMX-45P4ST with beryllium windows). Detailed descriptions of the detector characteristics are provided in [35]. The detector’s energy performance is delineated by a relative efficiency of 48% and an energy resolution, quantified as full width at half maximum (FWHM), at 2.16 keV at 1.33 MeV. The minimum detectable activity (MDA) of the system was estimated with a 95% confidence level. The spectral data were collected using the Ortec DSPEC-LF unit in conjunction with MCA Emulator software, Maestro-32, followed by analysis using the GammaVision Spectrum Analysis Software, version 7.01. Background spectra were obtained and subsequently subtracted to yield the measurement devoid of any additional background contributions. To ensure an adequate statistical sample, approximately 172,800 s (equivalent to 48 h) of counting time was allocated for the samples, while 259,200 s (equivalent to 72 h) was dedicated to background measurements. The analysis of the gamma-ray spectra from each sample involved consideration of transition energies of interest from both the ^238^U (63.2 keV and 92.5 keV for ^234^Th, 186 keV for ^226^Ra, 46.50 keV for ^210^Pb) and ^232^Th (911.1 keV and 968.9 keV for ^228^Ac) decay chains, as well as from ^40^K (1461 keV).

## 3. Results and Discussion

### 3.1. Gamma Spectrometry Results

**Figure 5:**
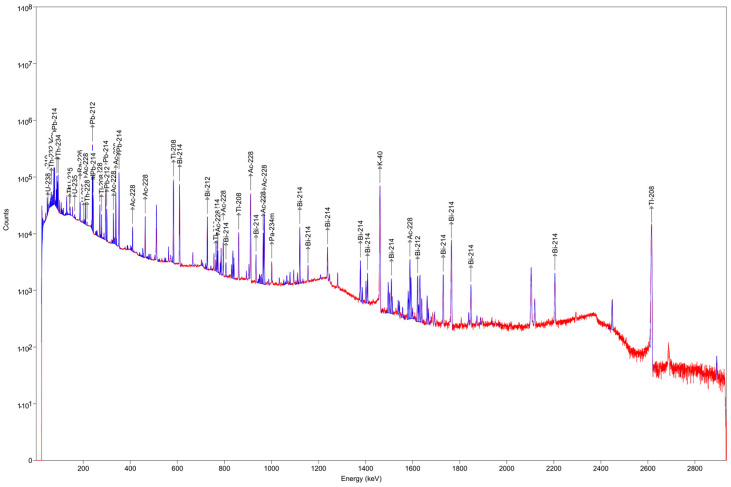
Spectrum from gamma spectrometry on grey tuff from Sant’Agata de’ Goti.

Three samples of volcanic gray tuff from Sant’Agata de’ Goti (Campania, Italy) were analyzed using gamma spectroscopy. The spectrum obtained is depicted in Figure 5; the activity concentrations of ^226^Ra and ^232^Th were determined by averaging the concentrations obtained from the radionuclides belonging to their respective decay chains of ^238^U and ^232^Th. The values of the activity concentrations of ^226^Ra and ^232^Th are, respectively, $C_{226_{Ra}}$ = 107 ± 4 Bq/kg and $C_{232_{Th}}$ = 186 ± 6 Bq/kg.

### 3.2. Alpha Spectrometry Results

**Figure 6:**
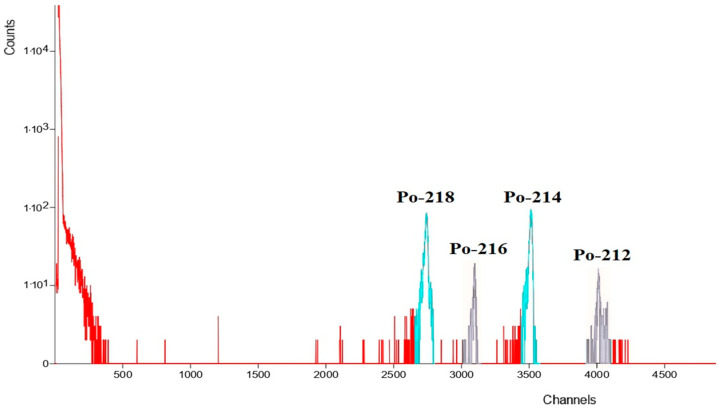
Spectrum from alpha spectrometry on gray tuff from Sant’Agata de’ Goti (in light blue ^222^Rn progenies, in gray ^220^Rn progenies).

Each powdered sample was placed into the collection chamber, and a measurement lasting 21 days was performed to allow for the attainment of secular equilibrium for ^226^Ra and ^232^Th with their emanated alpha decay daughters. The spectrum obtained is depicted in Figure 6.

**Figure 7:**
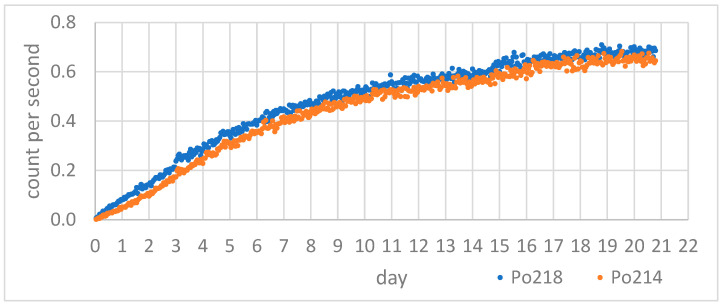
Counts per second of emanated ^218^Po and ^214^Po from a grey tuff sample from Sant’Agata de’ Goti village in Campania, Italy, for 21 days in the collection chamber.

**Figure 8:**
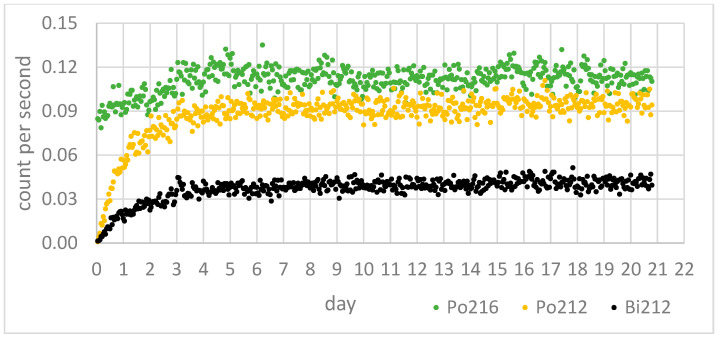
Counts per second of emanated ^216^Po, ^212^Po, and ^212^Bi from a grey tuff sample from Sant’Agata de’ Goti village in Campania, Italy, for 21 days in the collection chamber.

In Figure 7, the growth curves of ^218^Po and ^214^Po, progenies of ^222^Rn, can be observed for a sample from Sant’Agata de’ Goti. In Figure 8, the growth curves of the ^216^Po, ^212^Po, and ^212^Bi progenies of ^220^Rn can be observed for a sample from Sant’Agata de’ Goti. 

For the calculation of the emanation coefficient ($\eta_{222_{Rn}ema}$) and the exhalation rate ($E_{222_{Rn}}$) of ^222^Rn, the average counts per second (cps) of the ^218^Po after the equilibrium (obtained from the eighteenth day onward) were used in relations (1) and (5). For the calculation of the emanation coefficient ($\eta_{220_{Rn}ema}$) and the exhalation rate ($E_{220_{Rn}}$) of ^220^Rn, the cps of ^216^Po after equilibrium (obtained from the third day onward) was used in relations (3) and (6). 

In Table 1, the values of the emanation coefficient and the exhalation rate for both ^222^Rn and ^220^Rn are reported, derived from averaging measurements obtained from three different samples. These values were compared, always in Table 1, with those documented in the literature for similar types of grey tuffs from the Campania region [36]. The uncertainty of each value in Table 1 is about 10%. 

From the comparison between the emanation coefficients and exhalation rates measured in this study with the same parameters reported in the literature [36] for Campanian grey tuff, it emerged that Sant’Agata de’ Goti tuff exhibits high radon emanation and exhalation values for both isotopes. In particular, the emanation coefficient for ^220^Rn was about 10% lower than the highest value reported in the literature, while the emanation coefficient for ^222^Rn was higher by about 83%, the exhalation rate for ^222^Rn was higher by 110%, and the exhalation rate for ^220^Rn was higher by 65%.

No direct correlation between the activity concentration levels of ^226^Ra and ^232^Th and the emanation and exhalation capabilities of radon gas exists. The emanation and exhalation capacities of the matter are, in fact, due, in addition to the concentration of the radionuclides, to multiple petrographic characteristics, such as the size of the granules, the distribution of the radionuclides, and the porosity. The high values of the emanation coefficient and the exhalation rate found for the Sant’Agata de’ Goti tuff sample, even more than the high concentration values of the main radionuclides of natural origin (^226^Ra and ^232^Th) can mean high indoor radon concentration values.

## 4. Conclusions

The study highlights the urgent need for managing radon exposure in indoor environments to mitigate the substantial health risks associated with lung cancer carcinogenesis. The proposed metrological approach offers a robust framework for evaluating radon exhalation rates from several BMs by using an electrostatic collection chamber to measure the alpha-emitting progeny. The experimental setup was specifically tested to measure the exhalation rates of volcanic grey tuff with high radon activity concentrations in Sant’Agata de’ Goti village in Campania, Italy. The comparison between the emanation coefficient and exhalation rate values measured in this study and in the literature for Campanian grey tuff reveals how Sant’Agata de’ Goti tuff exhibits notably high radon gas emanation and exhalation properties. Aligned with legislative directives (European Directive 59/2013/Euratom and Italian Legislative Decree 101/2020) and the National Radon Action Plan, this research provides valuable insights for radon exhalation risk management in BMs, emphasizing the importance of identifying materials with heightened radon exhalation risks, thereby contributing to informed decision-making and regulatory compliance. These findings underscore the significance of continued research efforts to safeguard public health and reduce the incidence of radon-induced lung cancer due to exposure in indoor environments.

## Figures and Tables

**Table 1:** Comparison between emanation coefficients $\eta_{222_{Rn}ema}$ and $\eta_{220_{Rn}ema}$ as well as values of the exhalation rates $E_{222_{Rn}}$ and $E_{220_{Rn}}$ obtained in this work with similar grey tuff samples tuff from the Campania region reported in the literature [36].

	$\eta_{222_{Rn}ema}$ (%)	$E_{222_{Rn}}$ (10^−2^ Bq·kg^−1^·h^−1^)	$\eta_{220_{Rn}ema}$ (%)	$E_{220_{Rn}}$ (10^2^ Bq·kg^−1^·h^−1^)
Current work	22	17	8.2	6.88
Grey tuff (1)	8.0	4.7	4.1	1.92
Grey tuff (2)	12.1	8.1	9.0	4.16

## Data Availability

Data will be available upon request.
